# Superantigenic Activity of *emm*3 *Streptococcus pyogenes* Is Abrogated by a Conserved, Naturally Occurring *smeZ* Mutation

**DOI:** 10.1371/journal.pone.0046376

**Published:** 2012-10-01

**Authors:** Claire E. Turner, Mary Sommerlad, Karen McGregor, Frances J. Davies, Bruno Pichon, Deborah L. W. Chong, Leili Farzaneh, Matthew T. G. Holden, Brian G. Spratt, Androulla Efstratiou, Shiranee Sriskandan

**Affiliations:** 1 Department of Infectious Diseases & Immunity, Imperial College London, London, United Kingdom; 2 Department of Infectious Disease Epidemiology, Imperial College London, London, United Kingdom; 3 Respiratory and Systemic Infection Laboratory, The Health Protection Agency Centre for Infections, London, United Kingdom; 4 Pathogen Genomics, The Wellcome Trust Sanger Institute, Cambridge, United Kingdom; Universite Libre de Bruxelles, Belgium

## Abstract

*Streptococcus pyogenes* M/*emm*3 strains have been epidemiologically linked with enhanced infection severity and risk of streptococcal toxic shock syndrome (STSS), a syndrome triggered by superantigenic stimulation of T cells. Comparison of *S. pyogenes* strains causing STSS demonstrated that *emm*3 strains were surprisingly less mitogenic than other *emm-*types (*emm*1, *emm*12, *emm*18, *emm*28, *emm*87, *emm*89) both *in vitro* and *in vivo*, indicating poor superantigenic activity. We identified a 13 bp deletion in the superantigen *smeZ* gene of all *emm*3 strains tested. The deletion led to a premature stop codon in *smeZ*, and was not present in other major *emm-*types tested. Expression of a functional non-M3-*smeZ* gene successfully enhanced mitogenic activity in *emm*3 *S. pyogenes* and also restored mitogenic activity to *emm*1 and *emm*89 *S. pyogenes* strains where the *smeZ* gene had been disrupted. In contrast, the M3-*smeZ* gene with the 13 bp deletion could not enhance or restore mitogenicity in any of these *S. pyogenes* strains, confirming that M3-*smeZ* is non-functional regardless of strain background. The mutation in M3-*smeZ* reduced the potential for M3 *S. pyogenes* to induce cytokines in human tonsil, but not during invasive infection of superantigen-sensitive mice. Notwithstanding epidemiological associations with STSS and disease severity, *emm*3 strains have inherently poor superantigenicity that is explained by a conserved mutation in *sme*Z.

## Introduction

*Streptococcus pyogenes* is a major human pathogen, responsible for a wide spectrum of clinical manifestations. These range from non-invasive and self-limiting to severe, potentially lethal invasive infections complicated by streptococcal toxic shock syndrome (STSS). The development of STSS during *S. pyogenes* invasive infection is hypothesized to result from exposure to secreted streptococcal superantigens that leads to excessive proliferation of T cells, accompanying cytokine release, inflammation and tissue damage [1]. *S. pyogenes* can express several different superantigens (reviewed in [2]) that can vary in their potency, thus differences in mitogenicity within and between M/*emm*-types can be influenced by the complement of superantigen genes, as well as differences in expression or degradation [3]–[6]. SMEZ is the most potent streptococcal superantigen described, although produced in small amounts compared to other superantigens, [7]–[10] and the *smeZ* gene is present in the majority of *S. pyogenes* strains, with over 40 different alleles [8], [11].

In the UK, which has the highest measured rate of invasive *S. pyogenes* disease in Europe (3.33 per 100,000), infections with M/*emm*3 type *S. pyogenes* are independently associated with a three-fold increased risk of STSS, compared to patients infected with a reference strain group (M/*emm*28) that is represented by large numbers, and is associated with an average case fatality ratio [12]. Studies in the USA, Canada, and elsewhere in Europe have also highlighted an increased risk of death associated with M/*emm*3 *S. pyogenes* infection [13]–[16].

In this study, we compared superantigenicity of different *S. pyogenes emm-*types, using mitogenicity as a surrogate measure, and explored the hypothesis that superantigenic potency would be associated with STSS. Surprisingly we observed that *emm*3 strains had almost negligible mitogenicity compared to other *emm*-types. We sought to determine whether this was due to a recently identified 13 bp deletion within *smeZ* that yielded a premature stop codon and predicted inactive protein expression [17]. We went on to determine the frequency of the 13 bp deletion and the impact on superantigenic function in *emm*3 strains.

## Results

### *Emm3* Isolates from STSS Patients Lack Mitogenic Activity Compared with Other STSS Isolates, Both in vitro and in vivo

**Figure 1:**
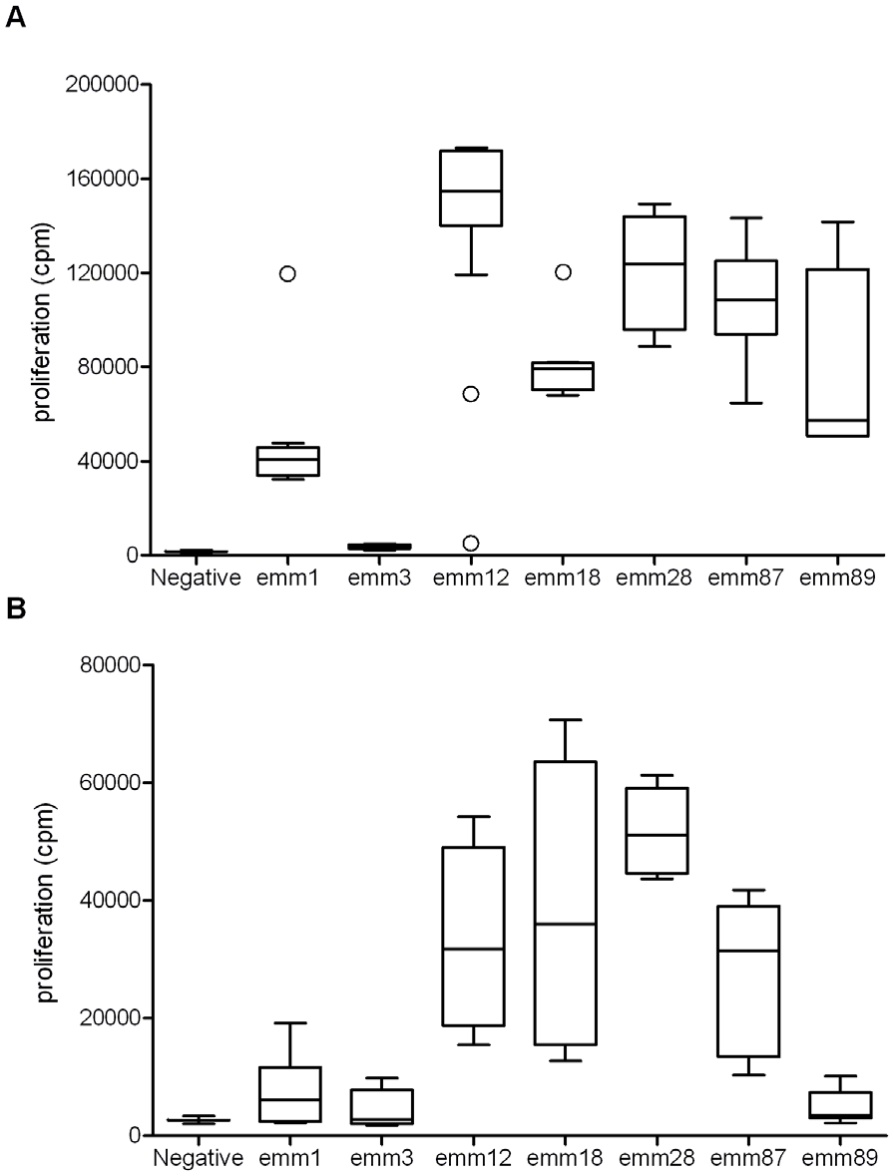
*Emm*3 STSS isolates show *emm*-type specific differences in overall mitogenicity. *A.*Human MNC proliferation response to culture supernatants from 63 *Streptococcus pyogenes* streptococcal toxic shock syndrome (STSS) isolates grouped by *emm*-type (numbers per group: *emm*1, n = 11; *emm*3, n = 12; *emm1*2 and *emm*87, n = 11; *emm*18, n = 5; *emm*28, n = 7; *emm*89, n = 6). Negative; tissue culture media (RMPI) alone. Outliers are represented as individual circles. Representative of 2 experiments performed on different donors. *B.* Human MNC proliferation response to sera obtained from CD1 mice 24 hours after being infected with one of three *S. pyogenes* strains representing each *emm*-type; two mice were infected per strain. Negative; uninfected mouse serum. Proliferative response is measured as counts per minute (cpm) of tritiated-thymidine uptake. Median and 5th, 25th, 50th, 75th, and 95th centiles shown.

The mitogenicity of a panel of 63 *S. pyogenes* isolates from cases of confirmed STSS was evaluated *in vitro* using human mononuclear cells (MNC) (Figure 1A). Strains represented the most common *emm-*types associated with STSS in the UK (*emm*1, n = 11; *emm*3, n = 12; *emm*12 and *emm*87, n = 11; *emm*18, n = 5; *emm*28, n = 7; *emm*89, n = 6). The complement of superantigens carried by each isolate was determined by PCR (Table S1). *Emm*3 and *emm*1 strains were markedly less mitogenic than other *emm-*types (Figure 1A) despite their reported association with STSS; this was reproducible even when MNC from alternative donors were used (not shown). In particular, some *emm*3 strains demonstrated mitogenic activity that was barely above control levels, despite the presence of 4 or 5 superantigen genes (*speA*, *smeZ, speG*, *ssa*, and *speK*).

To determine whether *emm*3 *S. pyogenes* strains might change in phenotype *in vivo*, during an infection, the superantigenic activity of *S. pyogenes* strains of different *emm*-types was compared *in vivo* using a murine intramuscular infection model; three representative strains were tested for each of the seven STSS *emm*-types. In this model, secreted bacterial superantigens enter the systemic circulation, hence mouse serum acquires mitogenic properties towards human lymphocytes [18]. Murine sera obtained 24 hours after onset of infection were tested for mitogenic activity with human MNC as a measure of released superantigen (Figure 1B). Similar to the *in vitro* results, *emm*3 and *emm*1 strains released markedly less mitogen into mouse serum than other *emm*-types, with the exception of *emm*89 strains. These differences were not attributable to differences in bacterial counts during infection; despite producing the lowest mitogenic activity in mouse serum, *emm*3, *emm*1 and *emm*89 strains demonstrated the greatest systemic spread from the site of infection (thigh tissue) to the spleen and liver (Table S2).

### Emm*3* Isolates Carry a Mutation in smeZ

**Figure 2:**
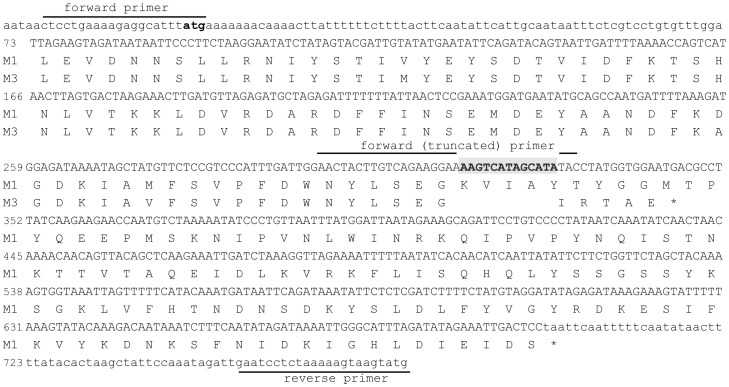
*emm*3 isolates have a 13 base pair (bp) deletion within the *smeZ* locus. Representation of the *smeZ* locus from *emm*1 (M1) and *emm*3 (M3) strains. Nucleotides 1 to 3 encode the start codon (shown in bold). From 73 bp of the nucleotide sequence, the amino acid sequence of the mature SMEZ protein is shown. *Emm*3 strains have a 13 bp deletion at 316 bp (highlighted by a shaded box) that results in a frameshift and a predicted premature stop codon after 86 amino acids (shown as *). The forward primer and the reverse primer amplify the full length *smeZ* locus. The 13 bp deletion was detected using a forward (truncated) primer that anneals specifically to the region containing the deletion.

Previously we had determined that five contemporary *emm*3 *S. pyogenes* strains carried a *smeZ* gene containing a 13 bp deletion at nucleotide position 316, that leads to a frame shift and a premature stop codon predicted to truncate SMEZ [17] (Figure 2). This *smeZ* allele (*smeZ59N*) was restricted to *emm*3 strains and was not detected in 44 other *emm*-types tested [17]. We detected the 13 bp *smeZ* deletion mutation by PCR in all 12 *emm*3 *S. pyogenes* strains associated with STSS (strains listed in Table S1). The same mutation was detected in 27/27 other *emm*3 *S. pyogenes* invasive diseasestrains submitted to the reference laboratory that included three of the most common MLST sequence types for *emm*3 strains; ST15, ST315, and ST406 (http://spyogenes.mlst.net/). The mutation was also identified in the two existing NCBI sequences for M3 *S. pyogenes* originating from the USA (MGAS315, GenBank accession number AE014074, [19]) and from Japan (SSI-1, GenBank accession number BA000034, [20]).

### Contribution of the 13 bp smeZ Mutation to Low Mitogenicity of emm3 Strains

**Figure 3:**
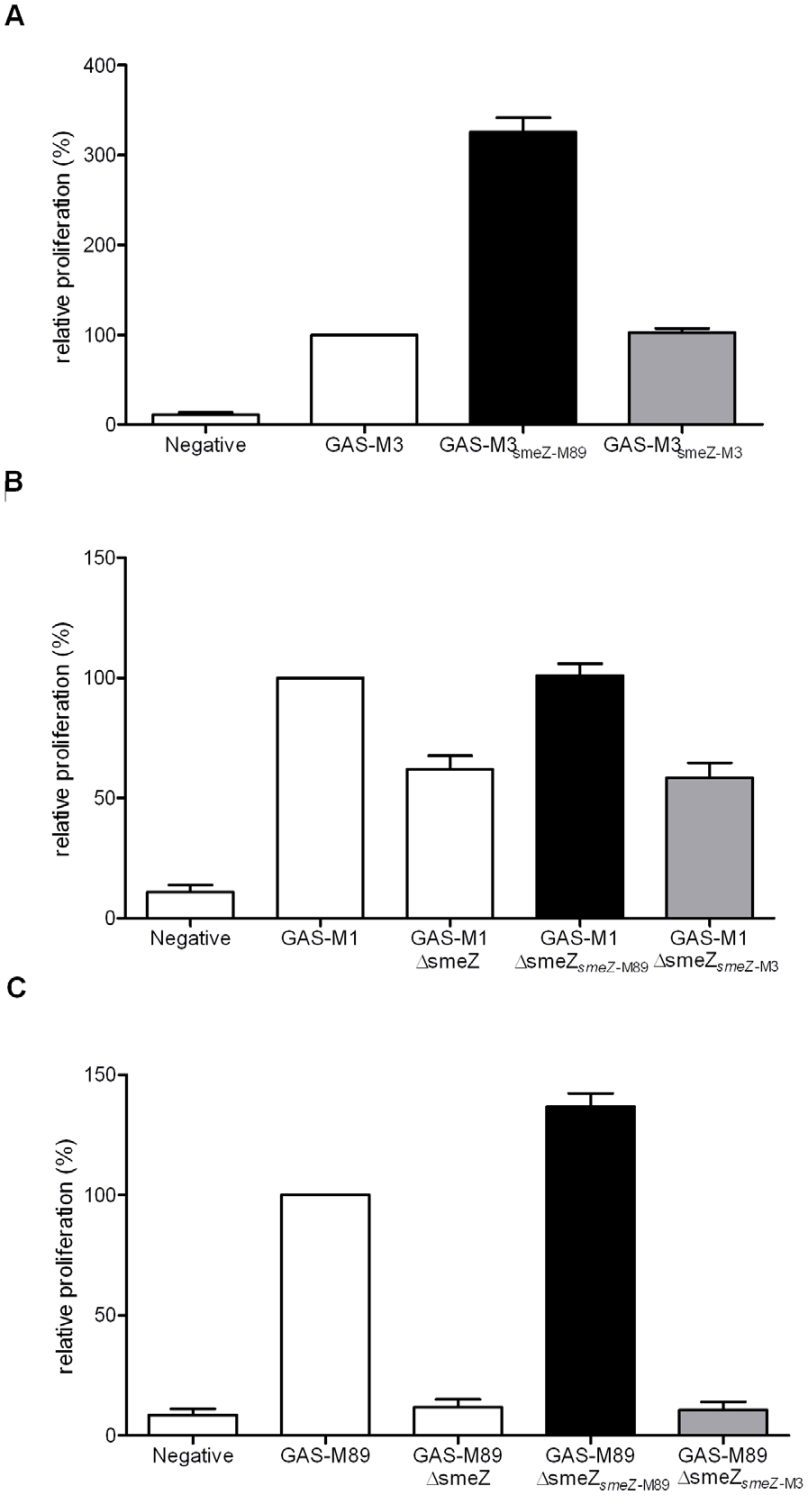
*emm*3 strains have low mitogenicity due to the mutation in *smeZ*. *A.* MNC proliferative response to culture supernatants from an *emm*3 isolate, GAS-M3 carrying the typical M3-*smeZ* with 13 bp deletion, GAS-M3 over-expressing the functional M89 form of SMEZ (GAS-M3*_smeZ_*_-M89_) and GAS-M3 over-expressing the M3-SMEZ (GAS-M3*_smeZ_*_-M3_). *B.* Experimental *smeZ* mutation in *emm*1 *S. pyogenes* (GAS-M1) reduced MNC proliferation (GAS-M1Δ*smeZ*). Proliferative response was restored when GAS-M1Δ*smeZ* over-expressed the functional M89 form of SMEZ (GAS-M1*_smeZ_*_-M89_) but not with M3-type form of SMEZ (GAS-M1*_smeZ_*_-M3_). *C*. A similar result was obtained using an *emm*89 strain (GAS-M89) with an experimental mutation in *smeZ* (GAS-M89Δ*smeZ*). Proliferation was restored when GAS-M89ΔsmeZ over-expressed the functional M89 form of SMEZ (GAS-M89*_smeZ_*_-M89_) but not with M3-type form of SMEZ (GAS-M89*_smeZ_*_-M3_). Negative; media alone.Proliferation was measured as counts per minute (cpm) of tritiated-thymidine uptake and the percentage proliferation for each strain was calculated relative to the wild type strain (GAS-M3, GAS-M1, GAS-M89 respectively). Data are mean (+standard deviation) of three measurements. Representative of two experiments performed using different donor MNC.

The mutation within the *smeZ*-M3 gene suggested that any protein, if expressed, would be non-functional, thus potentially accounting for the low mitogenic activity observed for *emm*3 strains. To confirm that the SMEZ-M3 protein is non-functional and that the low mitogenic activity of *emm*3 isolates was not accounted for by enzymatic degradation of SMEZ or interference with T cell mitogenicity, an *emm*3 isolate, GAS-M3 (with the *smeZ*-M3 gene) was transformed with a plasmid that conferred over-expression of either a functional SMEZ protein from an M89 strain (GAS-M3*_smeZ_*_-M89_) or the truncated SMEZ-M3 protein (GAS-M3*_smeZ_*_-M3_). Supernatant from strain GAS-M3*_smeZ_*_-M89_ increased proliferation of human MNCs by 3-fold compared to the parental (untransformed) *emm*3 isolate, GAS-M3 (Figure 3A), confirming that the M3 strain does not produce an inhibitor that interferes substantially with mitogen function. Supernatant from GAS-M3*_smeZ_*_-M3_ failed to enhance proliferation, confirming that SMEZ-M3 is non-functional as a mitogen. Transformation of GAS-M3 with the empty control plasmid (GAS-M3_control_) did not affect proliferation of human MNCs compared to untransformed GAS-M3 (not shown). Real time PCR analysis demonstrated that the transformed isolates expressed equivalent amounts of *smeZ* transcript from either plasmid (GAS-M3*_smeZ_*_-M89_∶163.0 copies of *smeZ* per 10,000 copies of *proS* and GAS-M3*_smeZ_*_-M3_∶183.8 copies of *smeZ* per 10,000 copies of *proS*); therefore differences in expression could not account for the differences in proliferation.

### The 13 bp Deletion Abrogates Function of SMEZ in Other Strain Backgrounds

To confirm that SMEZ-M3 cannot function as a mitogen in other *emm*-types, we used genetically modified *S. pyogenes* strains lacking functional *smeZ* and determined whether transformation with plasmids encoding *smeZ*-M3 or *smeZ*-M89 could complement the defect in mitogenicity. Disruption of *smeZ* in *emm*1 *S. pyogenes* (GAS-M1Δ*smeZ*) led to a ∼40% reduction in proliferation (Figure 3B); this strain also has genes encoding for other superantigens (*speA, speG, speJ*). A more dramatic reduction in mitogenicity (∼80%) was observed after disruption of *smeZ* in an *emm*89 strain (GAS-M89Δ*smeZ*) (Figure 3C), despite the presence of genes encoding for other superantigens (*speG*, *speH, speJ*) consistent with an earlier report [9]. Mitogenic activity was fully restored when GAS-M1Δ*smeZ* and GAS-M89Δ*smeZ* were transformed to over-express functional SMEZ-M89. Transformation of GAS-M1Δ*smeZ* (Figure 3B) and GAS-M89Δ*smeZ* (Figure 3C) to over-express the M3 form of SMEZ, however, failed to restore mitogenic potential, again confirming that SMEZ-M3 is not a functional mitogen.

### The 13 bp smeZ-M3 Deletion Reduces the Potential of emm3 S. pyogenes to Stimulatecytokine Production in Human Tonsil Cells

**Figure 4:**
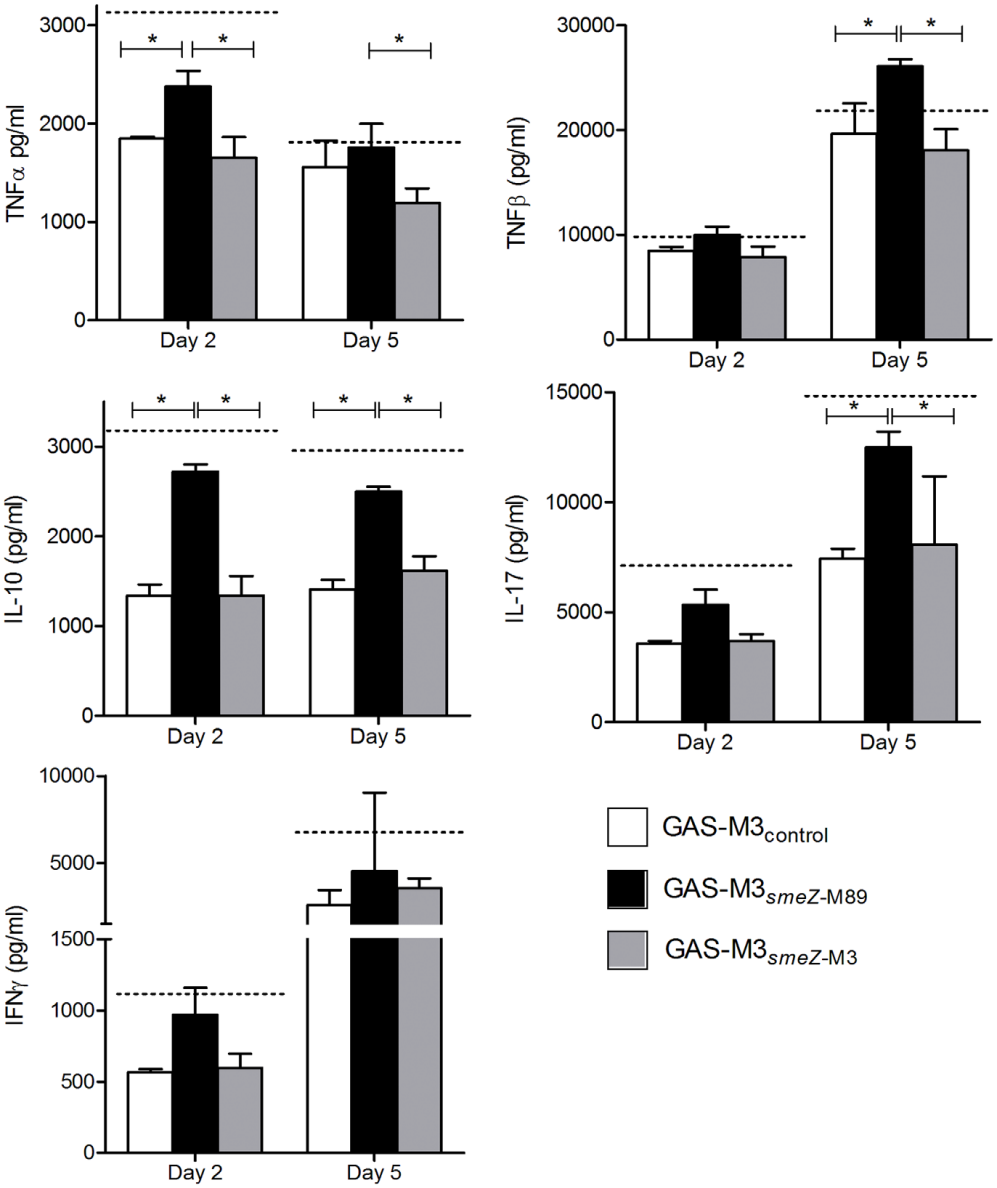
Functional SMEZ is required to stimulate production of cytokines from human tonsil cells. Human tonsil cell suspensions were cultured with bacterial cell-free culture supernatants from GAS-M3_control_(white bars), GAS-M3*_smeZ_*_-M89_ (black bars) and GAS-M3*_smeZ_*_-M3_ (gray bars). After 2 and 5 days incubation cell-free media were obtained from cultures and production of TNFα, TNFβ, IL-10, IL-17 and IFNγ were measured by ELISA. Horizontal dotted lines represent the mean level of cytokines produced after co-culture with bacterial cell-free culture supernatants from GAS-M1_control_ on day 2 and day 5. Mean (+ standard deviation) of three replicates measured in duplicate. Representative of two experiments performed on different donors. Statistical analysis was performed using ANOVA with Bonferroni multiple comparison.

Acute inflammation mediated by *smeZ* expression may be important for tonsillopharyngeal colonization of GAS [21]. To determine if the 13 bp mutation in *smeZ*-M3 might affect inflammation in tonsil, human tonsil cells were cultured with bacterial cell-free supernatants from GAS-M3_control_, GAS-M3*_smeZ_*_-M89_ or GAS-M3*_smeZ_*_-M3_. After two and five days of culture, production of TNFα, TNFβ, IFNγ, IL-10, IL-17, IL -5, and IL-12 were measured in supernatant, focusing on cytokines that have previously been identified as important in the human response to superantigens (Figure 4). In comparison to GAS-M3*_smeZ_*_-M3_, expression of functional SMEZ by GAS-M3*_smeZ_*_-M89_ enhanced the production of the modulating cytokine IL-10 at both times points, and also increased production of TNFα on day 2 and TNFβ and IL-17 on day 5 consistent with an effect on T cells. There was also a non-significant increase in IFNγ production by GAS-M3*_smeZ_*_-M89_. The levels of each cytokine induced by expression of functional SMEZ were comparable to those induced by GAS-M1_control_, which naturally expresses a functional SMEZ (Figure 4). In contrast, expression of SMEZ-M3 by GAS-M3*_smeZ_*_-M3_ did not affect cytokine production at any time point. Production of IL-5 and IL-12 were below the limit of detection. Experiments were repeated using a second tonsil donor with similar results (not shown).

### The 13 bp Deletion in smeZ-M3 Limits Only Interleukin-5 Production during Invasive Infection of Superantigen-sensitive Mice

To determine whether the non-functioning SMEZ-M3 affects cytokine production *in vivo* during GAS-M3 infection, we infected superantigen-sensitive HLA-DQ8 transgenic mice with GAS-M3*_smeZ_*_-M3_ or GAS-M3*_smeZ_*_-M89_ with a low intramuscular inoculum and measured both tissue and serum levels of cytokines after 24 h using a bead array.

**Figure 5:**
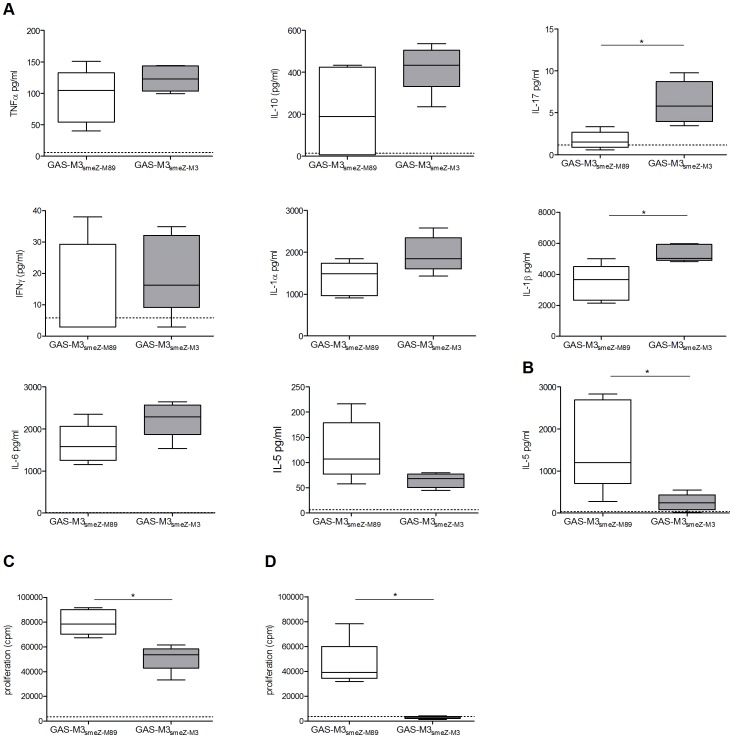
Functional SMEZ is not required for production of inflammatory cytokines in superantigen-sensitive mice. Groups of five superantigen-sensitive HLA-DQ8 female mice were infected for 24 hours intramuscularly (thigh) with 8×10^7^ CFU *emm*3 *S. pyogenes* strains; GAS-M3*_smeZ_*_-M89_ and GAS-M3*_smeZ_*_-M3_.*A.* Thigh tissue homogenate from mice infected with GAS-M3*_smeZ_*_-M3_, over-expressing SMEZ-M3 demonstrated significantly higher levels of IL-17 and IL-1β compared to GAS-M3*_smeZ_*_-M89_ infected thigh homogenate. Tissue from GAS-M3*_smeZ_*_-M3_ infection also demonstrated non-significant increases in IL-10 (*p = *0.059), IL-1α (*p* = 0.095) and IL-6 (*p* = 0.15). In contrast, GAS-M3*_smeZ_*_-M89_ had higher levels of IL-5 (*p* = 0.056). TNFα and IFNγ were similar in both groups. *B.* Mice infected with GAS-M3*_smeZ_*_-M89_ also had significantly higher levels of IL-5 in the circulating serum compared to GAS-M3*_smeZ_*_-M3_ infected mice. Horizontal dotted lines; lowest detectable level of each cytokine. Mitogenic activity was also measured in the thigh tissue homogenate (*C*) and serum (*D*) using human MNCs. GAS-M3*_smeZ_*_-M89_ demonstrated consistently higher mitogenic activity due to the over-expression of functional SMEZ compared to GAS-M3*_smeZ_*_-M3_. Horizontal dotted lines; proliferation level of uninfected control mouse thigh homogenate (*C*) or serum (*D*). Median and 5th, 25th, 50th, 75th, and 95th centiles are shown. For analysis, samples with unmeasurable levels of cytokine were assigned a value half the lowest detectable value. Proliferation was measured as counts per minute (cpm) of tritiated-thymidine uptake. Statistical analysis was performed using Mann-Whitney.

In contrast to *ex-vivo* stimulation of human tonsil cells and in contrast to previous studies using GAS-M89 [9], expression of functional SMEZ during intramuscular infection of HLA-DQ8 transgenic mice did not increase local tissue levels of TNFα, IL-10, IL-17 or IFNγ. In fact, mice infected with GAS that expressed the non-functional *smeZ* gene (M3*_smeZ_*_-M3_) had a trend for higher tissue cytokine levels (IL-17, IL-10, IL-1β) compared with mice infected with GAS expressing the functional *smeZ* gene (M3*_smeZ_*_-M89_) (Figure 5A). Most tissue cytokines were unaffected by the 13 bp deletion in SMEZ however (Figure S1).

The only cytokine influenced by the 13 bp deletion in *smeZ* was IL-5; IL-5 was higher in GAS-M3*_smeZ_*_-M89_ infection, not only at the site of infection but also in the serum (Figure 5A and B). Serum levels of other cytokines were no different between the two infected mouse groups (Figure S2).

To ensure that the two bacterial strains used had exhibited the expected phenotype *in vivo*, we assessed the mitogenic activity present in the thigh tissue and serum 24 h after infection. As expected, GAS-M3*_smeZ_*_-M3_ resulted in markedly lower mitogenic activity both at the local site of infection (thigh tissue homogenate, Figure 5C) and in the serum (Figure 5D) compared with GAS-M3*_smeZ_*_-M89_. The differences observed in mitogenicity and cytokine response were not due to measurable differences in bacterial burden as both groups had a similar bacterial load in thigh tissue after 24 hrs infection (GAS-M3*_smeZ_*_-M3_, median; 2×10^5^ CFU/mg range; 1×10^5^–3×10^5^ CFU/mg and GAS-M3*_smeZ_*_-M89,_ median; 1×10^5^ CFU/mg, range; 5×10^4^–5×10^5^ CFU/mg) and neither group demonstrated systemic spread.

## Discussion

Invasive infections due to *emm3 S. pyogenes* are widely associated with increased disease severity and, in the UK, with risk of STSS compared with other *emm*-types [12]–[15]. Intriguingly, *emm*3 *S. pyogenes* strains demonstrated very low mitogenic activity compared with other dominant *emm-*types that cause STSS; indeed, some *emm*3 strains had little mitogenic activity above background, which was surprising given the complement of 4–5 superantigen genes including *speA*. The observed deficiency in mitogenicity did not change even when measured *in vivo*, a setting where expression of superantigens can be enhanced. *Emm*3 strains carry a 13 bp deletion mutation in the *smeZ* sequence that changes the reading frame, resulting in a premature stop codon that is predicted to preclude expression of a functional protein [17], [22]. Thus, despite the presence of the *smeZ* gene, *emm*3 strains have no ability to produce active SMEZ superantigen. To a large extent this explains the failure of *emm*3 strains to match the mitogenic activity of other strains. The aberrant M3-*smeZ* was unable to restore mitogenicity when over-expressed in an *emm*3 strain, and also could not function when transferred to alternative *S. pyogenes emm*-types (*emm*1 and *emm*89). Strong mitogenic activity was associated with a functional *smeZ* gene and major reductions in mitogenic activity were associated with a non-functioning *smeZ* gene.

There are over 40 different *smeZ* alleles across the different *emm*-types sharing high pair-wise identity (94–99%) [8], [11], however the region surrounding the 13 bp deletion that occurs in the *emm*3 *smeZ* allele (*smez-59N*) is actually highly divergent across all the different alleles from different *emm*-types. Although no other non-*emm*3 alleles demonstrated deletions within this region, three *smeZ* alleles, *smez-6*, *smez-19* and *smez-23* from different *emm*-types had frame-shift single base pair deletions that occur closer to the N-terminus [8]. The 13 bp deletion in *smeZ* was found in all 39 *emm3* GAS strains tested, including strains from three different multilocus sequence types (ST15, ST315 and ST406). Further to this, the 13 bp deletion has also been found in all of 200 UK *emm*3 strains of ST15, ST315 or ST406, recently sequenced (unpublished). The deletion in *smeZ* was also present in *emm*3 genome strains from Japan (SSI-1, GenBank accession number BA000034) [20] and the USA (MGAS315, GenBank accession number AE014074) [19], both ST15. We were also able to identify the deletion in the Canadian *emm*3 strains, sequenced by Beres et al [23], by mapping the reads available on the short read archive (Project SRP000775) to the *smeZ* locus from the M1 strain MGAS5005. However, PCR analysis would be required to confirm that the deletion was identical to that found in UK *emm*3 as the region of divergence downstream of the deletion introduced ambiguity in the read mapping. Furthermore, the *emm*3 *smeZ* deletion is not a very recent event since we detected the same 13 bp deletion in a 1931 *emm*3 *S. pyogenes* puerperal sepsis isolate from Queen Charlottes Hospital, Hammersmith (Lynskey N. *et al*, unpublished). We postulate that the *smeZ* deletion is likely to have arisen early in the evolution of the *emm*3 lineage and the pseudogene conserved over decades.

Although *smeZ*-M3 encodes a mutated gene that lacks classical T cell mitogenic function, it remains possible that the truncated SMEZ-M3 has an alternative as-yet unrecognized function in GAS-M3 strains, such as, for example, interfering with the function of other superantigens or acting as a non-mitogenic activator of class II-positive cells. Results showed that *smez*-M3 is transcribed at a similar level to functional *smeZ*-M89 although we are unable to confirm if truncated SMEZ-M3 polypeptide is actually produced.

Interestingly, *emm*1 strains were also poorly mitogenic compared with other *emm-*types, both *in vitro* and *in vivo*. This was surprising since *emm*1 strains all carried *speA*, *speG*, and *speJ* as well as an intact *smeZ* gene. SPEA production is known to account for only a small proportion of *emm*1 mitogenic activity *in vitro* [24], although *emm*1 strains are able to upregulate production of superantigen during deep tissue infection *in vivo* [25], [26]. SPEJ is a superantigen that selectively expands T cells bearing the Vβ2 receptor [27]; since this is one of the largest subfamilies of human T cells, it was expected that *emm*1 strains might demonstrate heightened mitogenic activity compared with other *emm-*types. Nonetheless, targeted mutation of *smeZ* demonstrated clearly that SMEZ is an important component of *emm*1 mitogenic activity, similar to effects previously observed in an *emm*89 *S. pyogenes* strain [9]. We cannot exclude the possibility that *emm*1 and *emm*3 strains secrete proteins that are toxic to human T cells, although the ability of *smeZ*-M89 to confer mitogenicity suggests such an effect is not major. Degradation of superantigens by the streptococcal cysteine protease, SPEB may also contribute to reduced mitogenic activity of *S. pyogenes* [6] while the enhanced mitogenic properties of other *emm*-types may relate to synergistic actions of combinations of superantigens.

In addition to inducing T cell-derived cytokines, superantigens such as SMEZ can enhance TLR expression and signaling [9], [28]). The 13 bp deletion in *smeZ* limited pro-inflammatory cytokine production induced by GAS-M3 in tonsil cell suspension. We speculate that this could be of benefit to GAS-M3 as it may limit local inflammation in the pharynx, perhaps delaying the onset of symptoms and the innate or adaptive immune response to infection.

In contrast, the 13 bp deletion in *smeZ* did not appear to have marked effects on cytokine production elicited by GAS-M3 during *in vivo* invasive infection, with the exception of IL-5. Indeed the induction of IL-5 appeared to be largely dependent on SMEZ. Superantigen-induced expression of IL-5 *in vitro* has been previously reported [7], [29]. IL-5 is an eosinophilopoietic cytokine able to promote eosinophil maturation and survival; when activated, eosinophils can directly kill and enhance clearance of bacteria [30], [31]), thus a reduction in IL-5 may be of benefit to M3 GAS.

It was intriguing that the 13 bp deletion in *smeZ* did not have a wider impact on cytokines during invasive infection with GAS-M3 and, in certain cases, appeared to result in higher levels of cytokine despite very low levels of mitogenic activity in serum. This indicates that GAS-M3 has potent mechanisms of inducing cytokines during infection that are distinct from superantigen production, although we cannot exclude the additional possibility that a truncated form of SMEZ-M3 may play a role in enhancing inflammation. *Emm*3 *S. pyogenes* were recently shown to have acquired a novel prophage conferring the ability to produce a secreted phospholipase A2 which may play a role in lethal sepsis [32]. Clinical criteria that fulfill a diagnosis of septic shock due to *S. pyogenes* will normally also fulfill criteria required for STSS. Coupled with the many other virulence factors released by *S. pyogenes*, it is likely that STSS associated with *emm*3 *S. pyogenes* infection may not always require superantigen production.

## Materials and Methods

### Bacterial Strains

63 *S. pyogenes* isolates from patients with STSS meeting the criteria defined by the Working Group on Severe Streptococcal Infections [33], were provided by the Streptococcal Reference Laboratory, Health Protection Agency (HPA, London, UK), and represented *emm*-types that had been associated with STSS in at least five patients. These included 54 isolates from 2003–2004 (from a total of 167 STSS cases identified in England and Wales) and an additional 9 isolates from 2005–2006. *Emm-*typing was performed as previously reported [34]. *Emm*3 isolates from patients with confirmed invasive *S. pyogenes* infection without STSS were also provided by the HPA.

### PCR Analysis

The 13 bp deletion at position 316 of the *smeZ* nucleotide sequence of *emm*3 *S. pyogenes* strains is depicted in Figure 2. PCR primers were designed to amplify and distinguish the mutated *smeZ* gene, with the 13 bp deletion, from the non-mutated from, using a universal forward *smeZ* primer, Z1 (CTCCTGAAAAGAGGCTATTTATG), a universal reverse *smeZ* primer, Z2 (CATACTTACTTTTTAGAGGATTC), and an additional forward primer designed to anneal to the mutated (deleted) region, Z3 (AACTACTTGTCAGAAGGAATAC). Non-mutated *smeZ* yielded an amplification product of 796 bp with primers Z1 and Z2, while the mutated *smeZ* yielded a product of 474 bp with primers Z3 and Z2. Genotyping for other toxin genes was undertaken by multiplex polymerase chain reaction using the method previously reported [35] but analyzed by agarose gel electrophoresis. Primers that amplify the entire *smeZ* and *speJ* genes were additionally used, as allelic variation or pseudogenes can yield false negative and false positive results [36] (*smeZ-*F 5′-TAAAGGCTTTTTTGCTTGTTTCA, *smeZ*-R 5′-TTAGGAGTCAATTTCTATATCTAAATGCCC; *speJ*-F 5′-GATAGTGAAAATATTA, *speJ*-R 5′-TTATTTAGTCCAAAGG).

### Mitogenicity Assay

Total mitogenic activity of cell-free *S. pyogenes* supernatants was measured using a standard 72 hour human blood mononuclear cell (MNC) proliferation assay, as previously described [9]. Isolates were grown at 37°C to stationary phase in antibiotic-free tissue culture medium (RPMI 1640, Invitrogen, UK) containing fetal calf serum and L-glutamine [9], and bacterial cells were removed by centrifugation and 0.2 µM filtration. There were no significant differences in growth between *emm*-types. Proliferation of human MNCs was measured by tritiated-thymidine uptake after 72 hour co-incubation with 1∶100 dilution of cell-free, filter-sterilized bacterial supernatant in the absence of human serum as previously described [9]. Human MNCs were obtained from at least two different healthy donors. In addition to bacterial supernatant, the mitogenic activity of murine sera and thigh tissue homogenate were also tested at a 1∶100 dilution and co-incubated with human MNCs for 72 hours.

### Genetic Manipulation of emm1, emm3 and emm89 S. pyogenes Isolates

To determine whether mitogenic activity could be restored to an *emm*3 *S. pyogenes* strain (GAS-M3) with a functional copy of *smeZ*, the strain was transformed with the shuttle plasmid pDL278 carrying a copy of a functional *smeZ* gene from an *emm*89 strain with native promoter (pDL*smeZ-*M89) to generate strain GAS-M3*_smeZ-_*_M89_, using methods described previously [37]). As a control, GAS-M3 was also transformed with pDL278 carrying a copy of the *smeZ* locus with native promoter from *emm*3 *S. pyogenes* that contains the 13 bp deletion and premature stop codon (pDL*smeZ*-M3), to generate strain GAS-M3*_smeZ_*_-M3_. GAS-M3 also has genes for superantigens *speA, speG, speK* and *ssa.* To assess whether *smeZ*-M3 could function in an alternative *emm*-type *S. pyogenes* strain, the *smeZ* gene was firstly disrupted in *emm1 S. pyogenes* (GAS-M1) using methods described previously [9] to generate GAS-M1Δ*smeZ*. GAS-M1Δ*smeZ* was then transformed with either pDL*smeZ-*WT or pDL*smeZ*-M3. Additionally, these two plasmids were also used to transform a previously described *emm*89 strain (GAS-M89) with a disrupted *smeZ* gene (GAS-M89Δ*smeZ*) [9]. Where additional control strains were needed, parental GAS-M3, GAS-M1 and GAS-M89 strains were also transformed with the empty shuttle plasmid pDL278 to give GAS-M3_control_, GAS-M1_control_ and GAS-M89_control_. Strains are listed in Table 1.

**Table 1:** *S. pyogenes* strains used in this study.

Strain	Genotype
GAS-M1	*emm*1 strain (H305), wild type for *smeZ*
GAS-M1Δ*smeZ*	*smeZ* gene disruption in GAS-M1
GAS-M1Δ*smeZ_smeZ_*_-M89_	GAS-M1Δ*smeZ* over-expressing functional SMEZ from GAS-M89
GAS-M1Δ*smeZ_smeZ_*_-M3_	GAS-M1Δ*smeZ* over-expressing M3-type SMEZ (which contains a 13 bp deletion)
GAS-M1_control_	GAS-M1 transformed with empty shuttle plasmid
GAS-M89	*emm*89 strain (H293), functional *smeZ* (*smeZ*-13 allele)
GAS-M89Δ*smeZ*	*smeZ* gene disruption in GAS-M89 strain (H377)
GAS-M89Δ*smeZ_smeZ_*_-M89_	GAS-M89Δ*smeZ* over-expressing functional SMEZ from GAS-M89
GAS-M89Δ*smeZ_smeZ_*_-M3_	GAS-M89Δ*smeZ* over-expressing M3-type SMEZ (which contains 13 bp deletion)
GAS-M89_control_	GAS-M89 transformed with empty shuttle plasmid
GAS-M3	*emm3* strain with M3-*smeZ* (which contains a 13 bp deletion) (H471)
GAS-M3*_smeZ_*_-M89_	GAS-M3 over-expressing functional SMEZ from GAS-M89
GAS-M3*_smeZ_*_-M3_	GAS-M3 over-expressing M3-type SMEZ which contains a 13 bp deletion)
GAS-M3_control_	GAS-M3 transformed with empty shuttle plasmid

### Real Time PCR Analysis

*S. pyogenes* cells were obtained following supernatant collection for mitogenicity assay. RNA was extracted and real time PCR was performed as previously described [38] using SYBR Green Jumpstart Taq Readymix (Sigma-Aldrich, UK). Primers Z4 5′-TCCCTTCTAAGGAATATCTATAGTACGATTG and Z5 5′-TTCCAATCAAATGGGACGG were designed to amplify a 209 bp target region of *smeZ* common to all known alleles. The housekeeping gene *proS* was also amplified alongside *smeZ,* using primers *proS*-F 5′-TGAGTTTATTATGAAAGACGGCTATAGTTTC and *proS*-R 5′-AATAGCTTCGTAAGCTTGACGATAATC to generate a 93 bp product [39]. Copies of *smeZ* and *proS* transcripts were calculated against a standard plasmid containing the target region of the *smeZ* gene and the *proS* gene. Copies of *smeZ* transcript were then normalized against copies of *proS* transcript.

### Tonsil Cell Stimulation

Human tonsils were collected from adults undergoing routine tonsillectomy by the Imperial College Healthcare Trust Tissue Bank. Healthy tissue was cut into small sections which were then filtered through a 70 µM cell sieve with tonsil media (RPMI 1640, 10% fetal calf serum, 100 mM L-Glutamine, 250 iu/ml Penicillin, 250 µg/ml Streptomycin, 100 µg/ml Kanamycin, 2.5 µg/ml Amphotericin B; Invitrogen, UK) (method adapted from [40]). Cells were then washed twice before resuspending to 2×10^6^ cells per ml in tonsil media. In a 48 well plate format 1 ml of cells were incubated with 10% bacterial culture supernatant as for mitogenicity assays. After 48 hours (day 2) of media 500 µl was removed and stored at −20°C for cytokine analysis, and replaced with 500 µl fresh tonsil media. Cells were further cultured until day 5 when cells were removed by centrifugation and supernatant was stored at −20°C.

### Bacterial Infection

Female outbred CD1 mice were infected intramuscularly (thigh) with 10^6^–10^7^ CFU of *S. pyogenes*; three strains were used from each STSS-associated *emm*-type (*emm*1, 3, 12, 18, 28, 87 and 89) and two mice were infected per strain. After 24 hours of infection, mice were euthanized and blood was taken by cardiac puncture for CFU quantification and serum. Spleen, liver and infected thigh muscle were excised, individually weighed and homogenized in sterile PBS before plating on Columbia horse blood agar to quantify viable CFU. Remaining blood was centrifuged and serum was collected and stored at −20°C for testing mitogenic activity. Female humanized transgenic C57/BL/10HLA-DQ8 mice (kindly supplied by Daniel Altmann, Imperial College) were used as they are sensitive to superantigens including SMEZ [9]. Groups of 5 mice were infected intramuscularly (thigh) with 8×10^7^ CFU *emm*3 *S. pyogenes* strains; GAS-M3*_smeZ_*_-M89_ and GAS-M3*_smeZ_*_-M3_.After 24 hours, mice were euthanized and organs excised and plated as above. Both serum and infected thigh tissue homogenate were collected and stored for testing mitogenic activity and cytokine analysis using Luminex® (Invitrogen).

### Cytokine and Chemokine Measurement

TNFα, TNFβ, IFNγ, IL-10, IL-12,IL-17 (all R&D, UK), IL-5 (Peprotech, UK) and MCP-1 (Peprotech) were measured in human tonsil cell culture media using enzyme-linked immunosorbent assay (ELISA). Murine cytokine and chemokines were measured in infected serum or thigh homogenate using a mouse cytokine Luminex® 20-plex panel (Invitrogen) and analyzed on a Bio-Rad Bio-Plex 200 system. The cytokines and chemokines measured were as follows; TNFα,GMCSF, FGF, VEGF, IL-1α, IL-1β, IL-2, IL-4, IL-5, IL-6, IL-10, IL-12, IL-13, IL-17, MIP-1α, MCP-1, IFNγ, IP-10, MIG, and KC. For analysis, samples below the lowest level of detected were assigned a value half of the lowest measurable value.

### Ethics

The use of anonymized human tonsil tissue for research purposes was conducted within the remit of the Imperial HTA licence covering hospitals within ICHT and patients gave informed consent to use of tissues that would otherwise be discarded. Mice were used in accordance with UK Home Office guidance and subject to protocols set out in PPL 70/7379 approved by the Imperial College Ethical Review Process (ERP) panel.

### Statistical Analysis

All statistics were performed using non-parametric analysis with GraphPad Prism version 5.0 for Windows, GraphPad Software, San Diego California USA. *; p<0.05.

## Supporting Information

Figure S1 **Cytokine levels in thigh tissue homogenate from superantigen-sensitive HLA-DQ8 mice infected with GAS-M3** ***_smeZ_***_**-M89**_ **(White box-whisker) or GAS-M3** ***_smeZ_***_**-M3**_ **(Grey box-whisker).** Five mice per group were infected intramuscularly and after 24 hours infected tissue was removed and homogenated in sterile PBS. Cytokines were measured using Luminex®. Dotted horizontal line; lowest detectable level of each cytokine. For analysis, samples with undetectable levels of cytokine were assigned a value half the lowest detectable value.
(TIF)

Figure S2 **Cytokine levels in serum from superantigen-sensitive DQ8 mice infected with GAS-M3** ***_smeZ_***_**-M89**_ **(White box-whisker) or GAS-M3** ***_smeZ_***_**-M3**_ **(Grey box-whisker).** Five mice per group were infected intramuscularly and after 24 hours blood was removed by cardiac puncture. Cytokines were measured using Luminex®. Dotted horizontal line; lowest detectable level of each cytokine. For analysis, samples with undetectable levels of cytokine were assigned a value half the lowest detectable value.
(TIF)

Table S1 **Superantigen profile of each STSS isolate determined by PCR.**
(DOCX)

Table S2 **Bacterial colony forming units in spleen and liver following intramuscular infection with 7 different** ***emm*** **-types of** ***S. pyogenes.***
(DOCX)
