# Supplementary figures and images for: Superantigenic Activity of emm3 Streptococcus pyogenes Is Abrogated by a Conserved, Naturally Occurring smeZ Mutation
Source: PLoS One. 2012 Oct 1;7(10):e46376. doi: 10.1371/journal.pone.0046376 (PMC3462185; doi:10.1371/journal.pone.0046376)

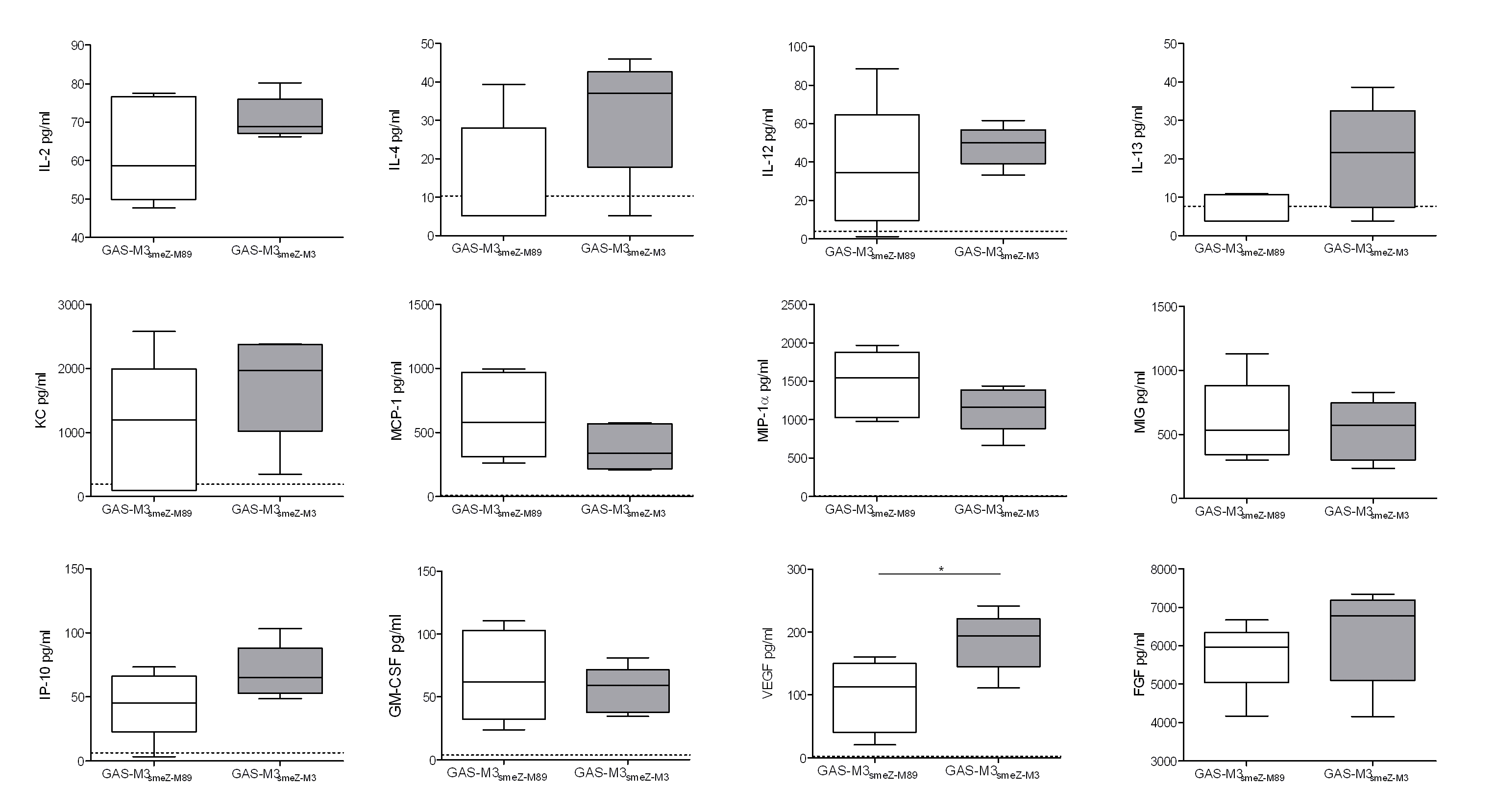

Supplement: Figure S1 — Cytokine levels in thigh tissue homogenate from superantigen-sensitive HLA-DQ8 mice infected with GAS-M3 smeZ -M89 (White box-whisker) or GAS-M3 smeZ -M3 (Grey box-whisker). Five mice per group were infected intramuscularly and after 24 hours infected tissue was removed and homogenated in sterile PBS. Cytokines were measured using Luminex®. Dotted horizontal line; lowest detectable level of each cytokine. For analysis, samples with undetectable levels of cytokine were assigned a value half the lowest detectable value. (TIF) [file pone.0046376.s001.tif]

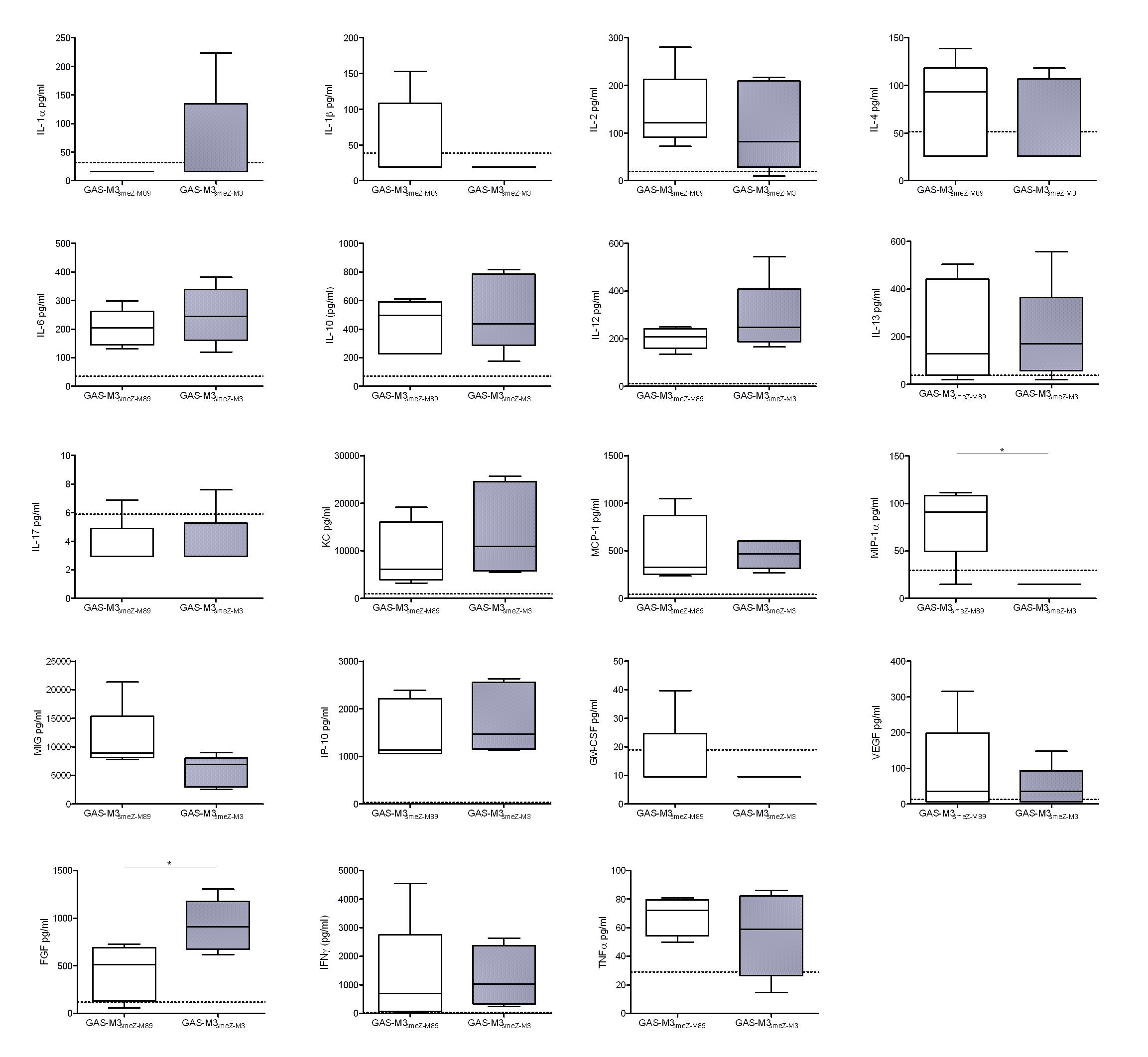

Supplement: Figure S2 — Cytokine levels in serum from superantigen-sensitive DQ8 mice infected with GAS-M3 smeZ -M89 (White box-whisker) or GAS-M3 smeZ -M3 (Grey box-whisker). Five mice per group were infected intramuscularly and after 24 hours blood was removed by cardiac puncture. Cytokines were measured using Luminex®. Dotted horizontal line; lowest detectable level of each cytokine. For analysis, samples with undetectable levels of cytokine were assigned a value half the lowest detectable value. (TIF) [file pone.0046376.s002.tif]
