# Supplementary material for: Superantigenic Activity of emm3 Streptococcus pyogenes Is Abrogated by a Conserved, Naturally Occurring smeZ Mutation
Source: PLoS One. 2012 Oct 1;7(10):e46376. doi: 10.1371/journal.pone.0046376 (PMC3462185; doi:10.1371/journal.pone.0046376)
Supplement: Table S1 — Superantigen profile of each STSS isolate determined by PCR. (DOCX) [file pone.0046376.s003.docx]

| M- type | *spe*A | *spe*C | *spe*G | *spe*H | *spe*I | *spe*J | *sme*Z | *ssa* | *spe*M | *spe*K | *spe*L | Lab No. |
| --- | --- | --- | --- | --- | --- | --- | --- | --- | --- | --- | --- | --- |
| 1 | + | - | + | - | - | + | + | - | - | - | - | H506 |
| 1 | + | - | + | - | - | + | + | - | - | - | - | H507 |
| 1 | + | - | + | - | - | + | + | - | - | - | - | H508 |
| 1 | + | - | + | - | - | + | + | - | - | - | - | H509 |
| 1 | + | + | + | - | - | + | + | - | - | - | - | H510 |
| 1 | + | - | + | - | - | + | + | - | - | - | - | H511 |
| 1 | + | - | + | - | - | + | + | - | - | - | - | H512 |
| 1 | + | - | + | - | - | + | + | - | - | - | - | H513 |
| 1 | + | - | + | - | - | + | + | - | - | - | - | H514 |
| 1 | + | - | + | - | - | + | + | - | - | - | - | H515 |
| 1 | + | - | + | - | - | + | + | - | - | - | - | H516 |
|  |  |  |  |  |  |  |  |  |  |  |  |  |
| 3 | + | - | + | - | - | - | + | + | - | - | - | H517 |
| 3 | + | - | + | - | - | - | + | + | - | + | - | H518 |
| 3 | + | - | + | - | - | - | + | + | - | + | - | H519 |
| 3 | + | - | + | - | - | - | + | + | - | + | - | H520 |
| 3 | + | - | + | - | - | - | + | + | - | + | - | H521 |
| 3 | + | - | + | - | - | - | + | + | - | + | - | H522 |
| 3 | + | - | + | - | - | - | + | + | - | - | - | H523 |
| 3 | + | - | + | - | - | - | + | + | - | + | - | H524 |
| 3 | + | - | + | - | - | - | + | + | - | + | - | H525 |
| 3 | + | - | + | - | - | - | + | + | - | + | - | H526 |
| 3 | + | - | + | - | - | - | + | + | - | + | - | H527 |
| 3 | + | - | + | - | - | - | + | + | - | + | - | H528 |
|  |  |  |  |  |  |  |  |  |  |  |  |  |
| 12 | - | + | + | + | - | - | + | - | - | - | - | H529 |
| 12 | + | - | + | - | - | - | + | + | - | - | - | H530 |
| 12 | - | + | + | + | - | - | + | + | - | - | - | H531 |
| 12 | - | + | + | + | - | - | + | + | - | - | - | H532 |
| 12 | - | + | + | + | - | - | + | - | - | - | - | H533 |
| 12 | - | + | + | + | - | - | + | - | - | - | - | H534 |
| 12 | - | + | + | - | - | - | + | + | - | - | - | H535 |
| 12 | - | + | + | + | - | - | + | + | - | - | - | H536 |
| 12 | - | + | + | + | - | - | + | + | - | - | - | H537 |
| 12 | - | + | + | + | - | - | + | + | - | - | - | H538 |
| 12 | - | + | + | + | - | - | + | + | - | - | - | H539 |
|  |  |  |  |  |  |  |  |  |  |  |  |  |
| 89 | - | + | + | + | - | - | + | + | - | - | - | H540 |
| 89 | - | - | + | - | - | - | + | - | - | - | - | H541 |
| 89 | - | - | + | - | - | - | + | - | - | + | - | H542 |
| 89 | - | - | + | - | - | - | + | - | - | - | - | H543 |
| 89 | - | - | + | - | - | - | + | + | - | - | - | H544 |
| 89 | - | + | + | - | - | - | + | - | - | - | - | H545 |
|  |  |  |  |  |  |  |  |  |  |  |  |  |
| 87 | - | + | + | - | - | - | + | + | - | - | - | H546 |
| 87 | - | - | + | - | - | - | + | + | - | - | - | H547 |
| 87 | - | - | + | - | - | - | + | + | - | - | - | H548 |
| 87 | + | + | + | - | - | - | + | + | - | - | - | H549 |
| 87 | + | + | + | - | - | - | + | + | - | - | - | H550 |
| 87 | + | + | + | - | - | - | + | + | - | - | - | H551 |
| 87 | + | + | + | - | - | - | + | + | - | - | - | H552 |
| 87 | + | + | + | - | - | - | + | + | - | - | - | H553 |
| 87 | + | + | + | - | - | - | + | + | - | - | - | H554 |
| 87 | + | + | + | - | - | - | + | + | - | - | - | H555 |
| 87 | + | + | + | - | - | - | + | + | - | - | - | H570 |
|  |  |  |  |  |  |  |  |  |  |  |  |  |
| 28 | - | + | + | - | - | - | + | - | - | - | - | H556 |
| 28 | - | + | + | - | - | - | + | - | - | - | - | H557 |
| 28 | - | + | + | - | - | - | + | - | - | - | - | H558 |
| 28 | - | + | + | - | - | - | + | - | - | - | - | H559 |
| 28 | - | + | + | - | - | - | + | - | - | - | - | H560 |
| 28 | - | + | + | - | - | - | + | - | - | - | - | H561 |
| 28 | - | + | + | - | - | - | + | - | - | - | - | H562 |
|  |  |  |  |  |  |  |  |  |  |  |  |  |
| 18 | + | + | + | - | + | - | + | - | + | - | + | H563 |
| 18 | + | + | + | - | + | - | + | - | + | - | + | H564 |
| 18 | + | + | + | - | + | - | + | - | + | - | + | H565 |
| 18 | + | + | + | - | + | - | + | - | + | - | + | H566 |
| 18 | + | + | + | - | + | - | + | - | + | - | + | H567 |
