# Supplementary material for: Superantigenic Activity of emm3 Streptococcus pyogenes Is Abrogated by a Conserved, Naturally Occurring smeZ Mutation
Source: PLoS One. 2012 Oct 1;7(10):e46376. doi: 10.1371/journal.pone.0046376 (PMC3462185; doi:10.1371/journal.pone.0046376)
Supplement: Table S2 — Bacterial colony forming units in spleen and liver following intramuscular infection with 7 different emm -types of S. pyogenes. (DOCX) [file pone.0046376.s004.docx]

|  | Spleen |  |  | Liver |  |
| --- | --- | --- | --- | --- | --- |
|  | Infected^a^ | Median (range) CFU/mg |  | Infected | Median (range) CFU/mg |
| *emm*1 | 4/6 | 41 (0-143) |  | 3/6 | 2.5 (0-16) |
| *emm*3 | 6/6 | 35 (3-276) |  | 5/6 | 2.5 (0-16) |
| *emm*12 | 1/6 | 0 (0-3) |  | 0/6 | 0 (0-0) |
| *emm*18 | 2/6 | 0 (0-16) |  | 0/6 | 0 (0-0) |
| *emm*28 | 0/6 | 0 (0-0) |  | 0/6 | 0 (0-0) |
| *emm*87 | 0/6 | 0(0-0) |  | 0/6 | 0 (0-0) |
| *emm*89 | 3/6 | 0.5 (0-156) |  | 1/6 | 0 (0-7) |

^a^number of mice with CFU detected in each organ
